# Characterisation of thigh-based electrocardiography (ECG) across different pathologies

**DOI:** 10.1038/s41598-026-46201-w

**Published:** 2026-03-28

**Authors:** Aline dos Santos Silva, Miguel Velhote Correia, Andreia Gonçalves da Costa, Rui J. Cerqueira, Hugo Plácido da Silva

**Affiliations:** 1 Institute for Systems and Computer Engineering, Technology, and Science (INESC TEC), Instituto de Telecomunicações (IT), Faculty of Engineering, University of Porto (FEUP), R. Dr. Roberto Frias, 4200-465 Porto, Portugal; 2 INESC TEC, Faculty of Engineering, University of Porto, R. Dr. Roberto Frias, 4200-465 Porto, Portugal; 3 OLI-Sistemas Sanitários, S.A., Travessa do Milão, 3800-314 Aveiro, Portugal; 4 Department of Surgery and Physiology, Faculty of Medicine, University of Porto, Porto, Portugal; 5 Department of Cardiothoracic Surgery, Hospital Universitário de São João, Porto, Portugal; 6 4LifeLAB, Porto, Portugal; 7 Instituto de Telecomunicações (IT), Instituto Superior Técnico, Department of Bioengineering, Av. Rovisco Pais, n. 1, Torre Norte-Piso 10, 1049-001 Lisbon, Portugal

**Keywords:** Cardiology, Computational biology and bioinformatics, Diseases, Medical research

## Abstract

Cardiovascular diseases remain the leading cause of morbidity and mortality worldwide. Continuous electrocardiographic (ECG) monitoring is essential for prevention and treatment, but conventional approaches based on the need for some voluntary action often limit comfort and adherence in long-term use. This study investigates the feasibility of acquiring ECG signals from a toilet seat interface embedding dry electrodes in the posterior thighs. A total of 30 hospitalised patients with diverse cardiovascular conditions–including arrhythmias, ischemic heart disease, heart failure, structural abnormalities, and aneurysms–were enrolled. Thigh-acquired ECGs were recorded simultaneously with conventional limb-lead signals and analysed for morphology, heart rate variability (HRV), and disease-related clustering. Thigh-based ECGs demonstrated clear P–QRS–T complexes with preserved morphology, allowing reliable extraction of mean templates and HRV metrics. The comparison between pathological and normal groups showed that post-surgical aortic repair patients had ECG profiles closest to the normal cluster; in contrast, aortic stenosis (AS) appeared most distant. HRV analysis revealed disease-specific autonomic patterns: patients with tricuspid or mitral involvement exhibited higher variability (SDNN up to 140 ms), whereas those with aortic valve disease presented markedly reduced parasympathetic indices (RMSSD and pNN50). Principal component analysis of multi-feature ECG data identified overlapping groups of Acute Coronary Syndrome, Unstable Angina and Ascending Aortic Aneurysm. At the same time, hierarchical clustering confirmed the distinct separation of conditions with severe hemodynamic disruption, such as PS and AS. These findings support the feasibility of unobtrusive thigh-based ECG monitoring via a toilet-seat interface, enabling reliable signal acquisition, HRV analysis, and preliminary patient stratification. This approach may lay the groundwork for future home-based cardiovascular screening and telemedicine applications.

## Introduction

Cardiovascular diseases (CVD) remain the leading cause of morbidity and mortality worldwide, driving the need for improved tools for early detection, continuous monitoring, and risk stratification^1^. Electrocardiography (ECG) remains the gold standard for assessing cardiac electrical activity. Still, conventional acquisition methods–such as limb- or chest-based electrodes–often face barriers to long-term adherence due to patient discomfort, technical complexity, and limited integration into daily life.

In recent years, there has been a growing interest in unobtrusive and ubiquitous approaches to physiological monitoring, including wearable devices, textile-based sensors, and smart household objects^2,3^. Toilet seat–based ECG acquisition represents a particularly promising solution, as it embeds electrodes in an object of routine daily use, enabling opportunistic monitoring without requiring additional effort from the patient^4–6^.

However, most studies to date have evaluated such systems either in healthy volunteers or under highly controlled experimental conditions, with limited validation in real-world clinical settings. This leaves a significant gap in the state of the art: the characterisation of unobtrusive ECG recordings in clinically heterogeneous populations, where a broad spectrum of cardiovascular conditions—often coexisting and influenced by medication, disease severity, and patient mobility–may substantially affect signal quality, morphology, and interpretability.

The present study addresses this gap by investigating the feasibility of acquiring clinically interpretable ECG signals via a toilet-seat interface in a diverse cohort of hospitalised patients with cardiovascular disease. The data were collected under opportunistic conditions that reflect realistic use scenarios rather than controlled laboratory protocols. In addition to qualitative and quantitative analyses of ECG waveform morphology, we include commonly used heart rate variability (HRV) metrics in an exploratory manner, aiming to illustrate what can be expected from these features in such short, unconstrained recordings, rather than providing a definitive assessment of autonomic function. Furthermore, dimensionality reduction and clustering techniques are applied to explore potential patterns and variability across diagnostic groups, and thigh-based recordings are compared with conventional limb-lead ECG when available.

The remainder of this paper is organised as follows: “Background” details the study population, acquisition setup, and signal processing methods; “Methods” presents the results on signal quality, HRV metrics, and clustering; “Results and discussion” discusses clinical implications, comparison with previous work, and limitations; and “Conclusion” concludes with future perspectives for unobtrusive cardiovascular monitoring.

## Background

Early detection and continuous monitoring of CVDs are essential for reducing morbidity and mortality, enabling timely interventions and improving patient outcomes^7,8^. Conventional diagnostic tools, such as the standard 12-lead ECG, are generally confined to clinical settings, limiting access for long-term or home-based monitoring^9^.

To address this limitation, non-intrusive monitoring systems integrated into everyday objects have been proposed, including toilet-seat ECG platforms^4–6^. These systems take advantage of natural user interaction with sanitary facilities to acquire cardiac signals without requiring voluntary action, offering a potentially scalable solution for passive monitoring.

Recent studies have demonstrated the clinical feasibility of this approach in healthy populations. For example, the COMMODE-seat from UMass Chan Medical School uses a smart toilet seat to monitor physiological signals, including heart rate and oxygen saturation, in individuals without known pathologies, aiming to improve health management^10^. Similarly, Casana developed the Heart Seat, a smart toilet seat capable of measuring cardiac signals, with clinical studies evaluating its performance mainly in healthy volunteers^11^.

To date, no published studies have systematically evaluated the acquisition and characterisation of ECG signals obtained via toilet seats in patients with diagnosed cardiovascular diseases. This gap highlights the need for investigations exploring the clinical applicability of this technology in pathological populations, which is the central objective of the present study.

Despite these advancements, the clinical characterisation of ECGs recorded on toilet seats in patients with specific cardiovascular pathologies remains limited. This study aims to fill this gap by assessing the feasibility and effectiveness of this technology in real clinical contexts.

## Methods

### Ethics statement

All experimental protocols and methods were conducted in accordance with the ethical principles for research involving human participants established by the Declaration of Helsinki. They were submitted to and approved by the Ethics Committee of the Cardiorespiratory Ward of Hospital São João, Porto, Portugal (approval number 315/23, dated September 2023).

An independent evaluation by the institution’s Data Protection Officer concluded that the potential risks to participants’ rights and freedoms were minimal. This assessment was based on multiple criteria, including the voluntary and informed nature of participant enrollment and the robust anonymisation procedures applied to the data. Each participant’s physiological signals were labelled with unique, study-specific codes that were not linked to personal identifiers, ensuring complete anonymity. All data were securely stored on encrypted institutional servers with daily backup protocols.

Written informed consent was obtained from all participants prior to inclusion in the study. Before participation, all individuals received a clear explanation of the study’s objectives, data acquisition procedures, estimated duration of participation (approximately 10 min), and confidentiality safeguards. Participation was entirely voluntary, and no monetary compensation was offered.

### Participants

The study explored two groups, namely subjects with and without diagnosed cardiovascular pathologies. Inclusion criteria for both groups required participants to be older than 18 years and able to sit independently on the experimental system. Exclusion criteria included the presence of implanted cardiac devices or the inability to provide informed consent.

A total of 86 individuals without cardiovascular pathology, all Portuguese nationals, voluntarily participated in this study. The group consisted of 50 females and 36 males, with an average age of $31.73 \pm 13.11$ years (ranging from 16 to 64 years), an average body weight of $66.89 \pm 10.70$ kg and an average height of $166.82 \pm 6.07$ cm.

Additionally, 30 individuals with diagnosed cardiovascular pathology, all Portuguese nationals, voluntarily participated in this study. The group included 14 women and 16 men, with a mean age of $63.48 \pm 14.00$ years (ranging from 34 to 82 years), a mean body weight of $70.44 \pm 13.40$ kg and a mean height of $161.81 \pm 9.34$ cm. The stratification of patients was carried out based on a broad range of diagnoses, including arrhythmias and heart failure, as well as other cardiovascular conditions such as cardiomyopathies, valvular diseases, and aneurysms.

The subjects with cardiovascular pathology were recruited through direct contact with the principal investigator at the Cardiorespiratory Ward of Hospital São João. All subjects had their condition diagnosed by a doctor specialising in the field, and only patients classified as stable at the time of recruitment were included.

The collection was intentionally diverse in terms of age and body composition to increase the generalisability of the results. The proportion of women and men was balanced across groups to the extent possible.

### Experimental setup and protocol

**Fig. 1:**
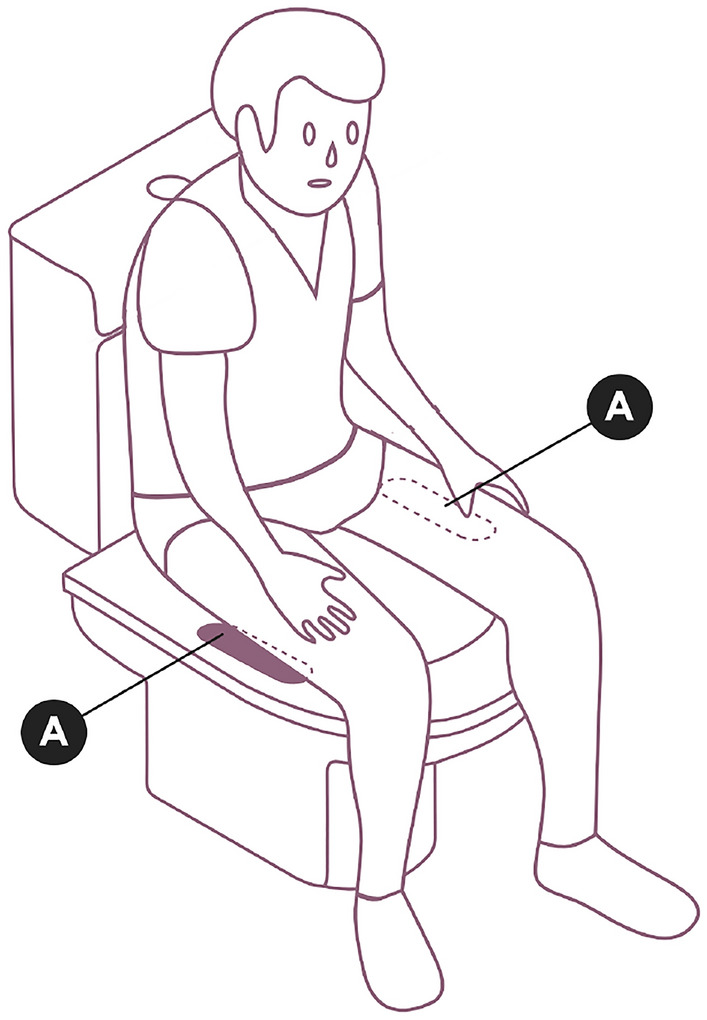
Schematic representation of sensor placement when using the smart toilet seat. A pair of ECG electrodes is positioned to contact the user’s thighs. Adapted from^5^.

Figure 1 illustrates the complete experimental configuration.

The ECG signals were collected using a custom-developed toilet seat system that incorporates a pair of dry electrodes for single-lead ECG recording. Further details regarding the system design and hardware configuration can be found in our previous work^5^. Signals were acquired at a sampling frequency of 1 kHz with 10-bit resolution.

Each recording session lasted up to two minutes, corresponding to the natural duration of toilet seat usage. As the acquisition followed an opportunistic paradigm, intended to simulate real-world deployment conditions realistically, not all subjects remained seated for the same amount of time. Consequently, recordings of variable duration were retained, provided they met predefined signal quality criteria. In the final dataset, the mean recording duration was 70.35 ± 49.65 seconds.

To ensure the reliability of ECG signals acquired from a non-conventional and noise-prone location, such as the thighs, an automated signal quality assessment procedure was applied before analysis. This procedure was adapted from our previously published processing pipeline^5^ and from established methodologies for ECG signal quality evaluation^12,13^. Given that motion artefacts, poor electrode contact, and analogue-to-digital converter (ADC) clipping primarily affect high-amplitude ECG components, signal quality was assessed at the beat level, with particular emphasis on the QRS complex.

R-peaks were first detected using a robust peak detection algorithm^14^. For each detected beat, saturation was evaluated by comparing the R-peak amplitude to the ADC dynamic range. A beat was classified as saturated if the R-peak amplitude reached or exceeded 95% of the full-scale ADC range, a threshold commonly used to identify clipping in physiological signals^15^. In addition, plateau-based saturation was assessed by analysing a temporal window of ±20 ms around each R-peak. Beats were flagged as corrupted if a flat or near-flat segment lasting at least 10 ms was observed within this window, defined by an absolute amplitude variation below 1% of the full-scale signal range. This criterion is widely employed to detect clipping and electrode overload in ECG recordings^13,16^.

Beyond saturation detection, overall signal quality was further evaluated using a signal-to-noise ratio (SNR) metric. The SNR was estimated by comparing the signal power within the ECG frequency band of interest (0.5–40 Hz) to the power outside this band, following standard ECG quality assessment approaches^12^. Recordings exhibiting a mean SNR below 5 dB were considered to have insufficient signal quality, consistent with thresholds reported in studies involving noisy and ambulatory ECG acquisitions^13^.

A recording was classified as invalid if more than 10% of the detected beats met one or more of the saturation or corruption criteria described above. Beat-level exclusion thresholds in the range of 10% are commonly adopted in ECG quality control pipelines to balance robustness and data retention^15,16^. Only recordings that satisfied all quality control criteria were retained for further analysis. After this exclusion step, a single valid recording per subject was included in the final dataset to ensure independence between samples.

All data processing and analysis were conducted using Python (v3.11.7). The BioSPPy package (v2.2.3) was employed for signal preprocessing, including band-pass filtering (0.5–40 Hz, 4th-order Butterworth), R-peak detection, beat segmentation, and feature extraction. The PyHRV library (v0.4.0) was used to compute HRV metrics. Unless otherwise specified, results are reported as mean ± standard deviation across participants.

### Diagnostic categories and ECG relevance

Tables 1 and 2 summarise the diagnostic categories included and the relevance of ECG for their clinical diagnosis, based on existing literature. All tables were reviewed and validated by two independent cardiologists.

**Table 1:** Diagnostic methods and ECG diagnosis for cardiovascular diseases with references.

No. samples	Code	Diagnosis	Diagnostic method	Diagnosis via ECG
2	AAA	Ascending aortic aneurysm	Imaging (CT/MRI)^17^	No
2	AI	Aortic insufficiency	Echocardiography^18^	Partial
2	AAR_M	Ascending aorta replacement + mediastinitis	Imaging + Surgical history^19^	No
7	AS	Aortic stenosis	Echocardiography^20^	Partial
2	AS_AAA	Aortic stenosis + ascending aortic aneurysm	Echocardiography^17^	Partial
1	ASD	Atrial septal defect	Echocardiography, cardiac MRI^21^	Rarely
4	CAD_ACS	CAD (acute coronary syndrome)	Coronary angiography^22^	Yes
2	CAD_UA	CAD (unstable angina)	Coronary angiography^23^	Yes
2	LAM	Left atrial myxoma	Echocardiography^24^	No
2	MI	Mitral insufficiency	Echocardiography^18^	Partial
1	PS	Pericardial stroke	Echocardiography, MRI^25^	No
1	RMS	Rheumatic mitral stenosis	Echocardiography^26^	Partial
2	TI_MI	Tricuspid and mitral insufficiency	Echocardiography^18^	Partial

**Table 2:** Cardiac effects and typical ECG findings in cardiovascular diseases with references.

Code	Cardiac effects	Typical ECG findings	Leads for observation
AAA	Aortic dilatation	Usually normal ECG^17^	None specific
AI	Aortic valve insufficiency	Left ventricular hypertrophy, possible ST changes^18^	V5-V6
AAR_M	Post-surgical changes, inflammation	Non-specific ST-T changes^19^	All leads
AS	Aortic stenosis	Left ventricular hypertrophy, strain pattern^20^	V1–V4
AS_AAA	Combined valve and aortic pathology	LVH, strain patterns^17^	V1–V6
ASD	Atrial septal defect	Right atrial enlargement, incomplete RBBB^21^	V1–V2
CAD_ACS	Ischemia and infarction	ST elevation/depression, Q waves^22^	V1–V6, II, III, aVF
CAD_UA	Ischemia without infarction	ST depression, T wave inversion^23^	V1–V6, II, III, aVF
LAM	Left atrial mass	Often normal ECG, possible atrial arrhythmias^24^	V1–V2
MI	Mitral insufficiency	Left atrial enlargement, possible atrial fibrillation^18^	I, II, V5–V6
PS	Pericardial stroke	Diffuse ST elevation, PR depression^25^	All leads
RMS	Mitral valve stenosis	Left atrial enlargement, atrial fibrillation^26^	II, V1
TI_MI	Right and left valve insufficiency	Atrial enlargement, possible arrhythmias^18^	II, V1–V2

The cardiac effects and typical ECG findings summarised in the table reflect a broad spectrum of cardiovascular and thoracic diseases with varying degrees of detectability through electrocardiography.

However, when recording ECG signals from a single lead placed on the thighs—as in this study—several challenges arise. Anatomical distance and altered conduction pathways can attenuate signal amplitude and alter waveform morphology compared to standard chest leads. While major abnormalities such as ventricular hypertrophy or significant arrhythmias might still be detectable, subtle ischemic changes or localised conduction delays may be masked or less discernible. Similar limitations have been reported in studies investigating alternative ECG lead placements^27^.

### Preprocessing and signal extraction

The raw ECG signals were filtered and segmented to extract individual cardiac beats. Using the BioSPPy library, each beat was delineated and resampled to 256 points to create a mean template per subject, ensuring participant comparability.

Features were extracted from each signal across multiple domains:

- Time-domain: heart rate (HR, BPM), RR intervals (ms), root mean square of successive differences (RMSSD, ms), standard deviation of normal-to-normal intervals (SDNN, ms), percentage of successive normal-to-normal intervals in an ECG that differ by more than 50 ms (pNN50, %);
- Frequency-domain: low frequency (LF, Hz), high frequency (HF, Hz), LF/HF ratio; spectral power was estimated using the Welch periodogram method with 256-sample Hanning windows and 50% overlap;
- Non-linear: entropy measures (SampEn) and detrended fluctuation analysis (DFA), which provide insights into the complexity and fractal properties of heart rate dynamics and are relevant for distinguishing pathological conditions from healthy variability;
- Morphological: wave durations in [ms] and amplitudes in [mV] of P, QRS, and T waves, as well as intervals (PR, QT). Two independent reviewers automatically extracted and visually inspected morphological parameters to ensure accuracy.

### Feature comparison and similarity analysis

Descriptive statistics were computed for each diagnostic group, and both intra-group and inter-group variability were analysed using Student’s *t*-tests or Mann–Whitney *U* tests depending on data normality. Given the limited sample sizes across several diagnostic categories, these statistical comparisons were interpreted cautiously and used primarily to highlight trends rather than to support definitive inferential conclusions.

To explore relationships and similarities among different cardiovascular and thoracic conditions, we applied Principal Component Analysis (PCA) and hierarchical clustering to ECG-derived features. These multivariate techniques were employed with a strictly exploratory objective, aiming to investigate how signals acquired from a non-conventional, thigh-based ECG configuration distribute in feature space under real-world clinical conditions. Visual tools such as PCA projections and hierarchical clustering dendrograms were used to qualitatively assess group separability, overlap, and dispersion rather than to define diagnostic boundaries.

It is important to emphasise that the pathological cohort comprised 30 hospitalised patients distributed across multiple diagnostic categories, reflecting the clinical heterogeneity of an opportunistic in-hospital acquisition scenario. Consequently, several subgroups contained a small number of subjects. For these groups, PCA projections, clustering outcomes, and group-level ECG templates are not intended to represent definitive or generalizable disease-specific morphologies. Instead, they serve to illustrate intra- and inter-group variability and to explore whether meaningful structure can still be observed despite limited sample sizes, medication effects, and diverse clinical presentations.

Euclidean distances between group-wise mean ECG templates were calculated to support the similarity analysis by providing a direct and interpretable measure of waveform dissimilarity in the time domain. While Euclidean distance may be less sensitive to subtle temporal misalignments than correlation-based metrics or dynamic time warping, it was intentionally selected for its simplicity, robustness, and compatibility with hierarchical clustering. In the context of this exploratory study, the use of Euclidean distance facilitates explainable comparison across groups while avoiding overfitting or excessive methodological complexity that would not be justified given the available sample sizes.

Overall, PCA, clustering, and template-based analyses were used as descriptive and hypothesis-generating tools, rather than as confirmatory methods, with the primary goal of assessing whether unobtrusively acquired thigh-based ECG signals preserve sufficient structure to support future large-scale and longitudinal studies.

## Results and discussion

### Similarity analysis between pathological groups

**Fig. 2:**
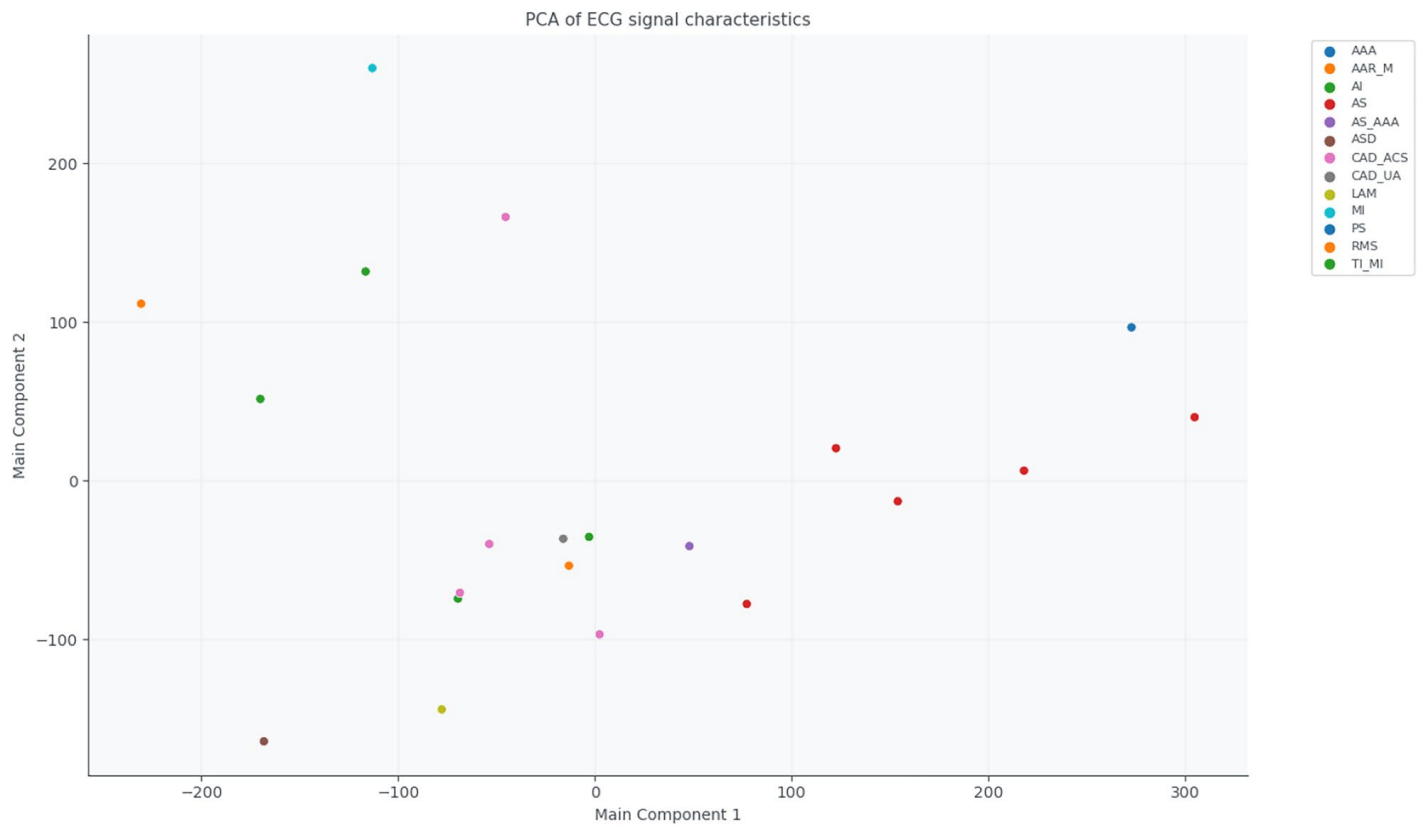
PCA biplot of ECG-derived features, showing the projection of each diagnostic group onto the first two principal components.

The PCA biplot (Fig. 2) shows the projection of each group onto two principal components. Groups such as RMS, AI, or AS_AAA form relatively compact clusters, indicating similar electrophysiological profiles. In contrast, groups like TI_MI, AAR_M, and MI are more dispersed, reflecting greater intra-group variability and clinical heterogeneity.

**Fig. 3:**
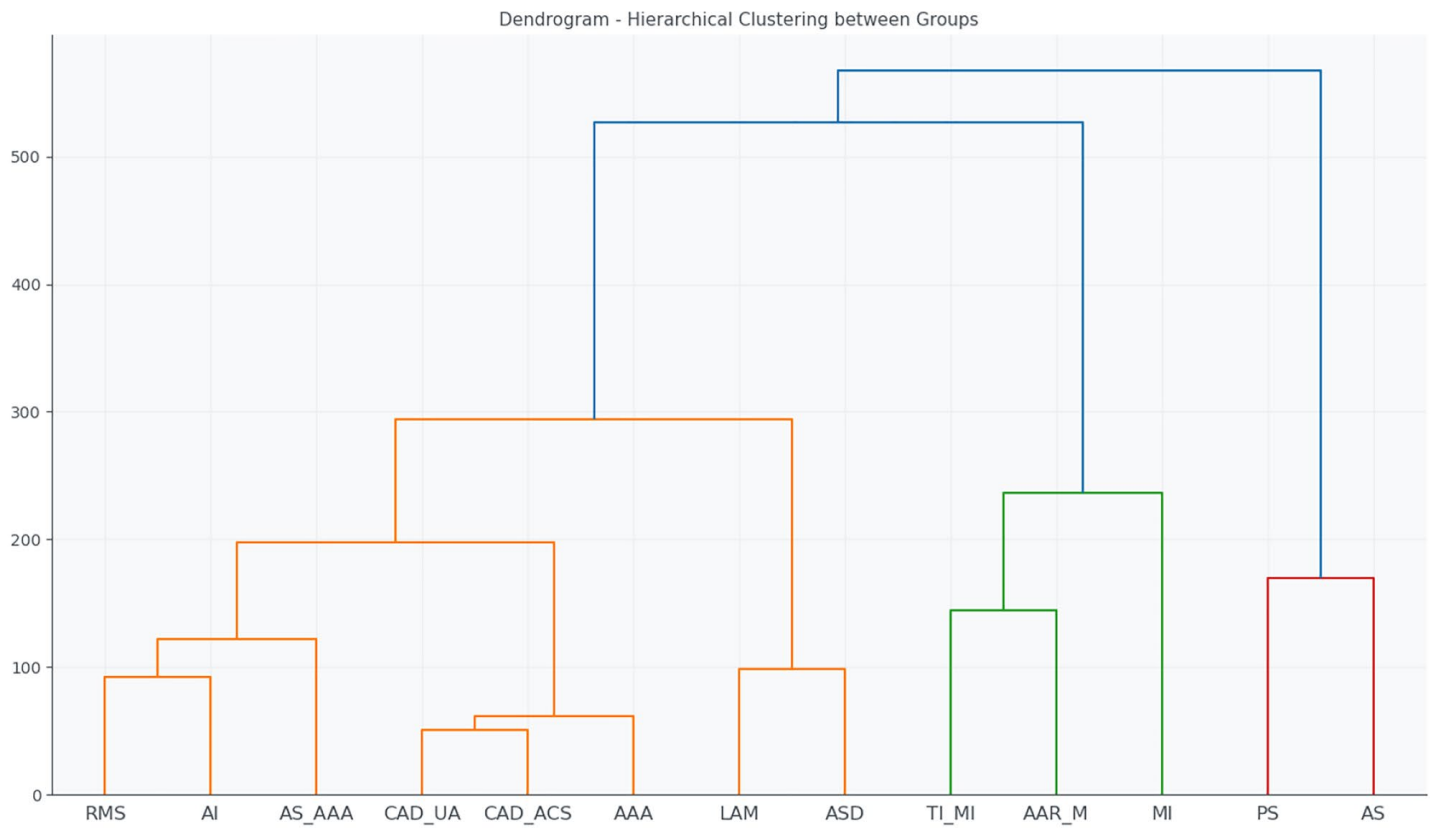
Dendrogram generated from hierarchical clustering of ECG signal features. The tree structure shows the similarity between patient groups, with shorter linkage distances indicating higher similarity in signal patterns.

Hierarchical clustering quantified these relationships, as shown in Table 3, which lists the 14 smallest Euclidean distances between the mean feature vectors of each group, as shown in the dendrogram of Fig. 3. Key observations include:

**Table 3:** Smallest Euclidean distances between diagnostic groups based on ECG features.

Group 1	Group 2	Distance
CAD_ACS	CAD_UA	50.75
AAA	CAD_UA	55.49
AAA	CAD_ACS	62.72
AI	RMS	92.48
ASD	LAM	98.34
AS_AAA	RMS	100.37
AS_AAA	CAD_UA	106.88
AS_AAA	CAD_ACS	114.27
CAD_ACS	RMS	115.67
CAD_UA	RMS	117.33
AAA	RMS	121.27
AAA	AS_AAA	123.55
AAA	LAM	124.23
CAD_UA	LAM	127.19

- CAD_ACS and CAD_UA (50.75): smallest distance, reflecting high similarity between acute and unstable coronary syndromes.
- AAA relative to CAD_UA and CAD_ACS (55.49 and 62.72): close proximity suggesting similar hemodynamic effects detectable on the ECG.
- AI-RMS (92.48) and ASD-LAM (98.34): more subtle electrophysiological affinities, possibly related to atrial remodelling or structural changes.

These results demonstrate that, even from an unconventional acquisition method such as the toilet seat, ECG signals retain sufficient diagnostic information to differentiate major cardiovascular pathologies. The hierarchical clusters reflect shared pathophysiological mechanisms such as ischemia, ventricular overload, or structural remodelling.

### Comparative analysis of mean ECG templates and HRV metrics

For interpretability, diagnoses were grouped into four clinically meaningful categories, allowing morphological and autonomic differences to be highlighted:

1. Coronary artery disease and comorbidities (CAD_ACS, CAD_UA, TI_MI): representing ischemic conditions with varying severity and autonomic modulation.
2. Aortic valve pathologies (AS, AI, AS_AAA): reflecting pressure and volume overload effects on cardiac electrophysiology.
3. Mitral and tricuspid valve diseases (MI, RMS, TI_MI): highlighting conduction alterations due to atrioventricular involvement.
4. Non-valvular structural abnormalities (AAA, LAM, ASD): representing heterogeneous anatomical impacts outside the valves.

**Fig. 4:**
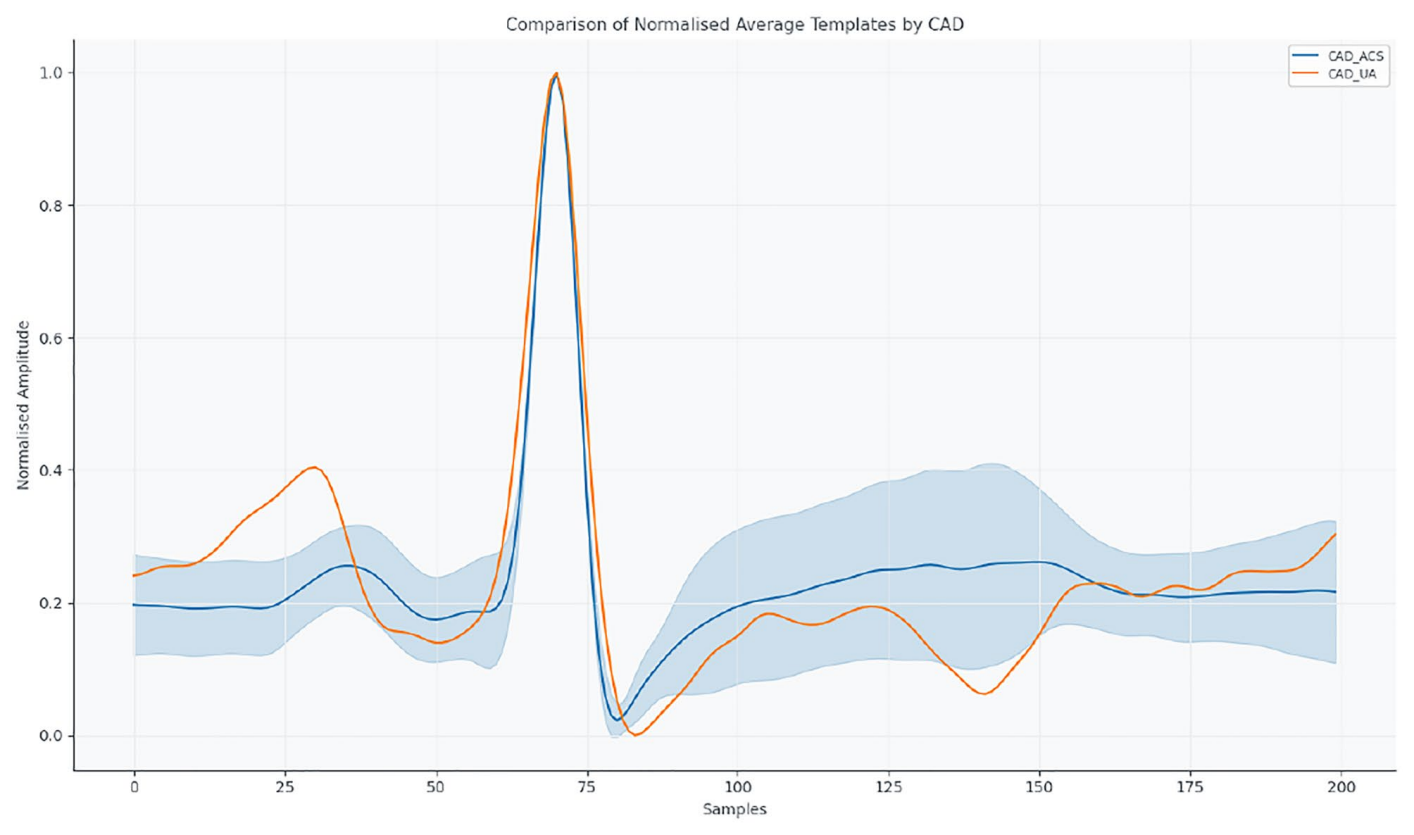
Mean normalized ECG templates for CAD_ACS and CAD_UA.

Figure 4 shows coronary artery disease presentations (CAD_ACS and CAD_UA), highlighting morphological differences in QRS and T waves.

**Fig. 5:**
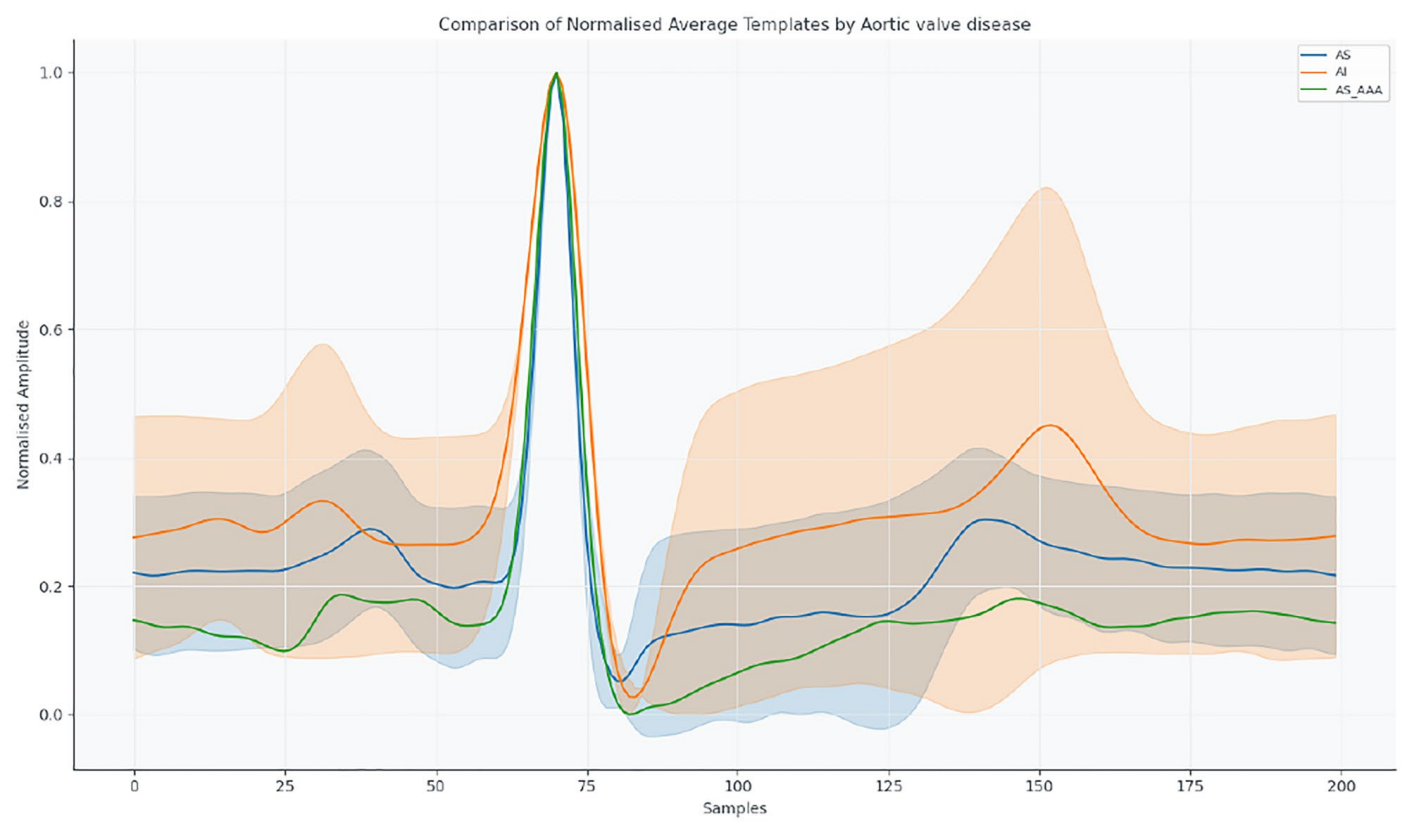
Mean normalised ECG templates for AS, AI, and AS_AAA.

**Fig. 6:**
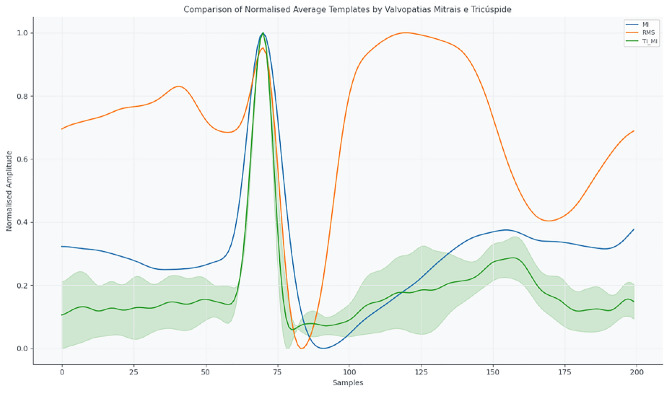
Mean normalised ECG templates for MI, RMS, and TI_MI.

Figures 5 and 6 display templates for aortic and mitral/tricuspid valve pathologies, respectively, illustrating conduction and repolarisation changes.

**Fig. 7:**
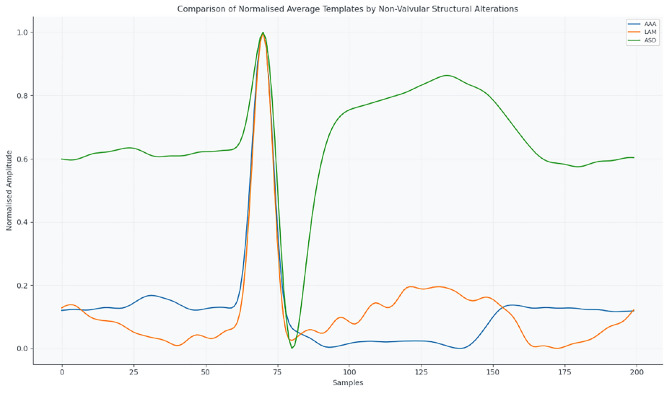
Mean normalised ECG templates for structural non-valvular abnormalities: AAA, LAM, ASD.

Figure 7 presents structural non-valvular alterations (AAA, LAM, ASD), reflecting the effect of anatomical modifications on ECG morphology.

Analysis of HRV metrics (MeanNN, SDNN, RMSSD, pNN50) across these categories reveals distinct autonomic profiles. Coronary patients retain partial parasympathetic modulation, aortic valve diseases display sympathetic predominance, and structural or extracardiac conditions exhibit high interindividual variability, reflecting heterogeneous anatomical and functional effects.

**Fig. 8:**
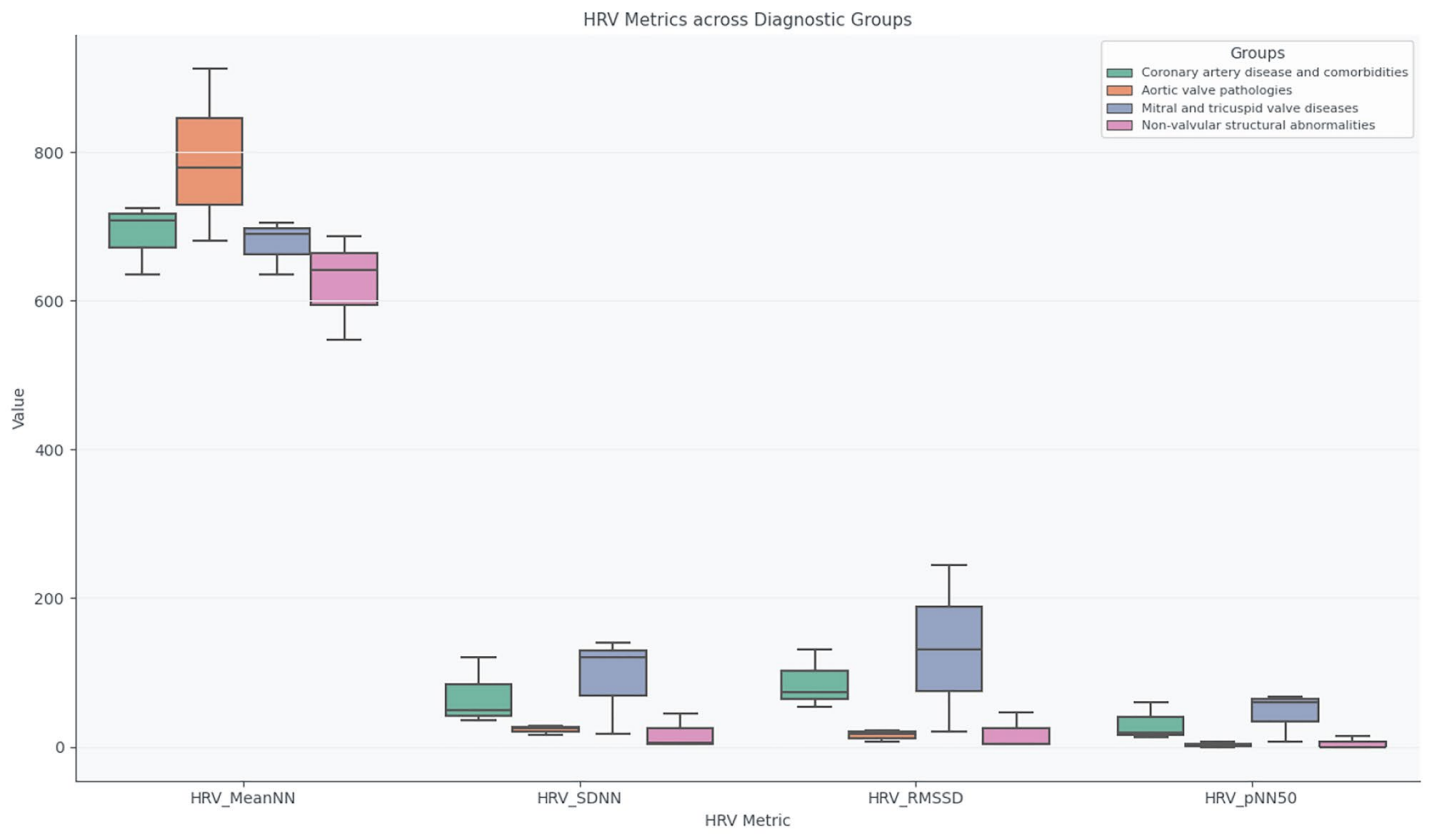
Boxplots of HRV metrics (MeanNN, SDNN, RMSSD, pNN50) across the four diagnostic categories.

Figure 8 summarises HRV metrics across the four diagnostic categories, illustrating the differences in autonomic regulation.

Overall, coronary artery disease and comorbidities show moderate HRV with preserved parasympathetic activity; aortic valve pathologies demonstrate reduced HRV, consistent with sympathetic predominance; mitral and tricuspid valve diseases present variable HRV depending on compensatory autonomic responses; and structural non-valvular abnormalities exhibit heterogeneous profiles, reflecting diverse anatomical impacts on cardiac function.

### Comparison between normal and pathological groups

Table 4 summarises a qualitative comparative analysis between pathological groups and the normal group, based on their relative position in the hierarchical clustering of mean ECG feature vectors. The visual distance to the normal centroid was inferred from the linkage distance at which each pathological group merges with the normal cluster in the dendrogram, reflecting global similarity in the multidimensional feature space. Intra-group dispersion represents the observed variability within each pathological group, capturing physiological heterogeneity across subjects. The estimated similarity combines both centroid proximity and intra-group dispersion, providing an overall interpretative measure of how closely each pathological condition resembles normal ECG dynamics.

Within this framework, post-surgical aortic replacement (AAR_M) exhibits high similarity to the normal group, indicating minimal residual deviation and low internal variability. In contrast, PS and AS show pronounced deviations and low similarity, consistent with their substantial structural and functional impact on cardiac electrophysiology.

**Table 4:** Qualitative similarity between pathological groups and the normal group based on visual distance.

Code	Visual distance to normal	Intra-group dispersion	Estimated similarity	Remarks
AAR_M	Very close	Low	High	Minimal deviation; post-surgical but near-normal features
ASD	Close	Moderate	Moderate/High	Slight displacement with moderate variability
AAA	Close	Low	High	Structural dilation with limited feature spread
MI	Moderately close	Moderate	Moderate	Infarction effect visible, but not extreme
TI_MI	Moderately distant	High	Moderate/Low	Mixed dysfunction profile increases dispersion
CAD_UA	Distant	High	Low	Ischemic features with large variability
CAD_ACS	Distant	High	Low	Acute phase characteristics create heterogeneity
AS_AAA	Distant	Moderate	Low	Combined deformities increase deviation
LAM	Moderately distant	Moderate	Moderate/Low	Tumor-induced displacement of cardiac dynamics
PS	Very distant	High	Very low	High deviation due to acute obstruction
RMS	Moderately distant	Moderate	Moderate/Low	Remodeling alters baseline structure
AS	Very distant	Moderate	Very low	Severe valvular dysfunction dominates features

**Fig. 9:**
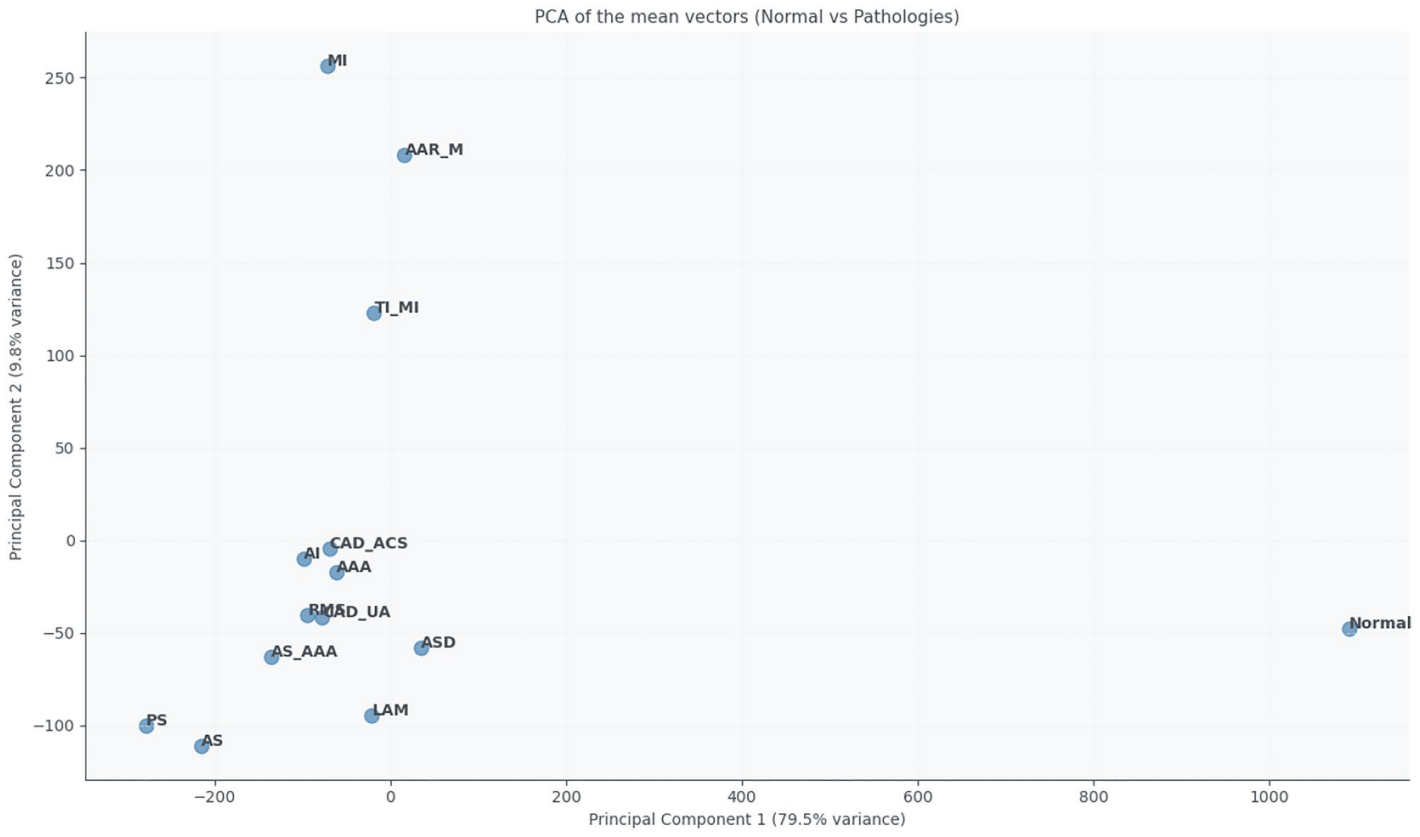
PCA of the mean feature vectors for the normal and pathological groups.

**Fig. 10:**
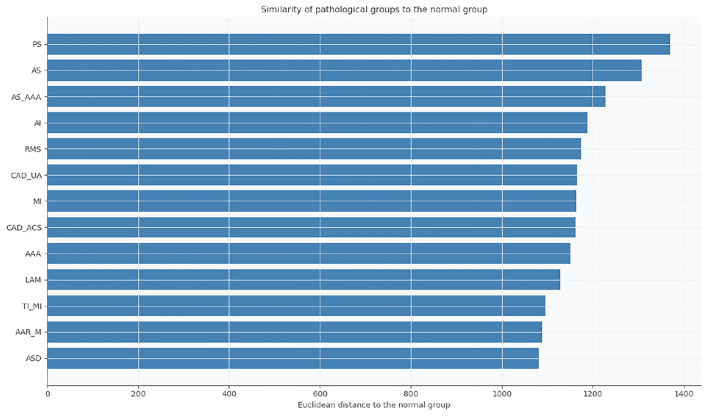
Euclidean distances between the mean feature vectors of each pathological group and the normal group.

Figures 9 and 10 summarise the similarities and differences between the normal and pathological groups based on the extracted ECG features. The PCA projection (Fig. 9) reduces the multidimensional feature space into two principal components that capture the most significant variance in the data. PC1 and PC2 together explain the majority of variability, allowing visualisation of inter-group relationships. In this projection, the normal group forms a distinct cluster, clearly separated from pathological categories such as PS, AS, and CAD_UA, indicating substantial deviations in cardiac electrophysiology. Other groups, including AAR_M and MI, partially overlap with the normal cluster, suggesting that their cardiac function and morphology are relatively preserved or only mildly altered.

**Fig. 11:**
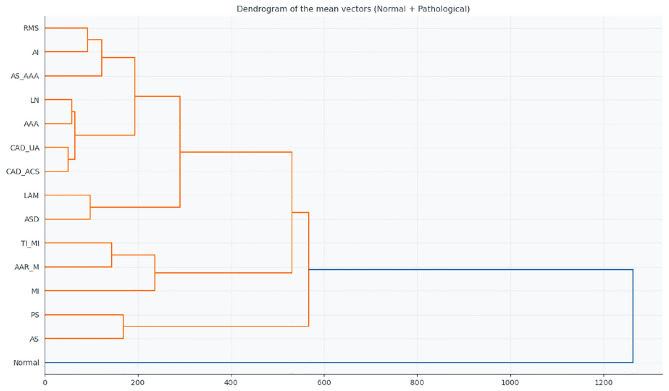
Hierarchical clustering dendrogram of the mean feature vectors for the normal and pathological groups.

The hierarchical clustering dendrogram (Fig. 11) corroborates these observations by grouping the most similar profiles based on the Euclidean distances of their mean feature vectors. AAR_M and MI are clustered near the normal group, while PS and AS are positioned farther away, reflecting more severe or complex structural and functional abnormalities.

Finally, the Euclidean distance analysis (Fig. 10) provides a quantitative measure of deviation from normality. Groups such as AAR_M, ASD, and AAA exhibit the smallest distances to the normal profile, indicating preserved cardiac electrophysiology, whereas PS and AS show the largest distances, consistent with significant pathological alterations.

Taken together, these analyses indicate that conditions involving localised defects or surgically corrected abnormalities maintain profiles closer to normal. In contrast, severe obstructive or combined pathologies produce the most significant deviations in ECG-derived features, highlighting their distinct electrophysiological signatures.

### Physiopathological interpretation and clinical implications

These results indicate:

1. Clusters reflect shared pathophysiological mechanisms: proximity indicates common autonomic, hemodynamic, or structural influences.
2. HRV complements morphological interpretation: metrics such as SDNN, RMSSD, and pNN50 capture disease-specific autonomic modulation.
3. Proximity to normal profile: guides patient stratification and phenotyping, potentially useful for longitudinal or remote monitoring.
4. Unconventional acquisition is valid: single-lead ECG signals capture diagnostically relevant information.

*Limitations* include small sample size, single-lead ECG acquisition, and potential medication effects. Future studies should validate findings in larger cohorts, explore sub-clustering in heterogeneous groups, and evaluate applications in digital health monitoring.

## Conclusion

This study demonstrates that cardiovascular and thoracic conditions present distinct patterns in ECG morphology and feature-space representations. By integrating principal component analysis, hierarchical clustering, radar plot visualisation, and comparisons of mean ECG templates, we identified internal relationships and clustering tendencies among diagnostic groups. These analyses revealed pathophysiological proximities, such as shared signatures among ischemic and thoracic conditions, while also highlighting distinct profiles in complex structural anomalies, including pulmonary stenosis (PS) and aortic stenosis (AS).

Notably, ECG signals were acquired noninvasively using a smart toilet seat with the contact interface on the thighs. Despite this unconventional acquisition site, the extracted features preserved clinically meaningful distinctions between pathological groups, confirming the robustness of feature-based ECG analysis. Groups such as AAR_M and AAA demonstrated high similarity to the normal cardiac profile, suggesting minimal functional impact or effective post-surgical correction. In contrast, PS and AS consistently appeared as outliers, reflecting severe structural and hemodynamic deviations.

The clustering of CAD_ACS, CAD_UA, and AAA suggests overlapping electrophysiological patterns, potentially arising from shared autonomic dysregulation or systemic vascular strain. These findings highlight the potential of similarity metrics to differentiate disease states, reveal latent comorbidities, and support differential diagnosis when conventional ECG interpretation is inconclusive.

From a clinical and technological perspective, the results underscore the promise of low-burden, contact-based ECG monitoring systems—such as toilet-integrated platforms—for longitudinal population screening, real-world cardiac monitoring, and integration into intelligent clinical decision-support tools.

An essential limitation of this study is the relatively small sample size per diagnostic category, due to the difficulty of recruiting patients with highly specific cardiac pathologies under controlled acquisition conditions. Despite this, the methodology’s exploratory and innovative nature provides a strong foundation for future research. Subsequent studies should focus on expanding the dataset, investigating intra-group variability, incorporating longitudinal or pre- and post-surgical data where possible, and validating the approach in diverse clinical contexts.

In conclusion, this study confirms the feasibility and clinical relevance of ECG feature analysis from non-traditional acquisition sites. The observed clustering patterns provide a foundation for developing unobtrusive, intelligent diagnostic tools that leverage morphological and statistical similarities across various cardiovascular conditions.

## Data Availability

This study was reviewed and approved by the Ethics Committee of the Cardiorespiratory Ward of Hospital São João, Porto, Portugal, under approval number 315/23 (September 2023). Owing to this ethical approval and the sensitive nature of the data, ECG signals from patients with cardiac pathologies cannot be publicly shared without prior authorization from the committee. However, the dataset corresponding to participants without diagnosed cardiac conditions is publicly available on PhysioNet: Silva, A. S., Plácido da Silva, H., Correia, M., Gonçalves da Costa, A. C., & Laranjo, S. (2026). tOLIet: Single-lead Thigh-based Electrocardiography Using Polimeric Dry Electrodes (version 1.0.1). PhysioNet. RRID:SCR_007345. https://doi.org/10.13026/411k-1476.
